# SEDENTARY BEHAVIOR DIFFERENTIALLY INFLUENCES COGNITIVE HEALTH IN OLDER ADULTS AT DIFFERENT TIMESCALES

**DOI:** 10.1093/geroni/igad104.3584

**Published:** 2023-12-21

**Authors:** Tian Qiu, Daniel Elbich, Jessie Alwerdt, Jonathan Hakun

**Affiliations:** Pennsylvania State University, University Park, Pennsylvania, United States; The Pennsylvania State University, Hershey, Pennsylvania, United States; Pennsylvania State University, University Park, Pennsylvania, United States; Pennsylvania State University, University Park, Pennsylvania, United States

## Abstract

The negative health effects of sedentary behavior have received increased attention over the past decade. Whether sedentary behavior negatively influences cognitive health at any timescale remains poorly understood. Our study sought to determine whether sedentary behavior negatively affects cognitive health throughout the adult lifespan at multiple timescales (acute within-day, day-to-day, cross-sectionally). We examined ambulatory assessment data generated by an adult lifespan sample (N=161; age range:19-88 years; 84% F) where cognition was repeatedly assessed via ultra-brief cognitive assessments of processing speed and working memory in an ecological momentary assessment (EMA) framework (6 assessments/day for 14 days). Activity was measured continuously via thigh-worn ActivPAL monitors. ActivPAL and EMA data were combined and modeled in a multi-level (3-level; moments within days within persons) isotemporal substitution modeling framework. Increased sedentary behavior was associated with acute decreases in processing speed for older participants (65+ years of age) and substitution modeling indicated that exchanging bouts of walking or standing for sedentary time would result in short-term improvements. In contrast, a consistent result across younger and older adults indicated that days with generally more sedentary time (as well as lying and standing) were associated with overall better processing speed and working memory capacity. No cross-sectional associations were observed. A lack of cross-sectional associations between sedentary behavior and cognitive performance was consistent with previous studies. Our current results extend previous findings by showing that sedentary behavior might influence cognitive performance differently at different timescales in younger and older adults.

